# MHz data collection of a microcrystalline mixture of different jack bean proteins

**DOI:** 10.1038/s41597-019-0010-0

**Published:** 2019-04-03

**Authors:** Marie Luise Grünbein, Johan Bielecki, Alexander Gorel, Miriam Stricker, Richard Bean, Marco Cammarata, Katerina Dörner, Lars Fröhlich, Elisabeth Hartmann, Steffen Hauf, Mario Hilpert, Yoonhee Kim, Marco Kloos, Romain Letrun, Marc Messerschmidt, Grant Mills, Gabriela Nass Kovacs, Marco Ramilli, Christopher M. Roome, Tokushi Sato, Matthias Scholz, Michel Sliwa, Jolanta Sztuk-Dambietz, Martin Weik, Britta Weinhausen, Nasser Al-Qudami, Djelloul Boukhelef, Sandor Brockhauser, Wajid Ehsan, Moritz Emons, Sergey Esenov, Hans Fangohr, Alexander Kaukher, Thomas Kluyver, Max Lederer, Luis Maia, Maurizio Manetti, Thomas Michelat, Astrid Münnich, Florent Pallas, Guido Palmer, Gianpietro Previtali, Natascha Raab, Alessandro Silenzi, Janusz Szuba, Sandhya Venkatesan, Krzysztof Wrona, Jun Zhu, R. Bruce Doak, Robert L. Shoeman, Lutz Foucar, Jacques-Philippe Colletier, Adrian P. Mancuso, Thomas R. M. Barends, Claudiu A. Stan, Ilme Schlichting

**Affiliations:** 1 Max Planck Institute for Medical Research, Jahnstrasse 29, 69120 Heidelberg, Germany; 2 European XFEL GmbH, Holzkoppel 4, 22869 Schenefeld, Germany; 3 Department of Physics, UMR 625, UBL, University of Rennes 1, 35042 Rennes, France; 4 Deutsches Elektronensynchrotron DESY, Notkestraße 85, 22607 Hamburg, Germany; 5 BioXFEL STC, 700 Ellicott Street, Buffalo, NY 14203 USA; 6 ARC Centre of Excellence for Advanced Molecular Imaging, La Trobe Institute for Molecular Science, La Trobe University, Melbourne, VIC 3086 Australia; 7 Center for Free-Electron Laser Science, Deutsches Elektronensynchrotron, Notkestraße 85, 22607 Hamburg, Germany; 8 Laboratoire de Spectrochimie Infrarouge et Raman, CNRS, UMR 8516, Université de Lille, 59000 Lille, France; 9 Institut de Biologie Structurale, Université Grenoble Alpes, CEA, CNRS, 38044 Grenoble, France; 10 Biological Research Centre (BRC), Hungarian Academy of Sciences, Temesvári krt. 62, Szeged, 6726 Hungary; 11 University of Southampton, SO17 1BJ Southampton, United Kingdom; 12 Department of Physics, Rutgers University Newark, 101 Warren Street, Newark, NJ 07102 USA

**Keywords:** Nanocrystallography, Enzyme mechanisms, Proteins

## Abstract

We provide a detailed description of a serial femtosecond crystallography (SFX) dataset collected at the European X-ray free-electron laser facility (EuXFEL). The EuXFEL is the first high repetition rate XFEL delivering MHz X-ray pulse trains at 10 Hz. The short spacing (<1 µs) between pulses requires fast flowing microjets for sample injection and high frame rate detectors. A data set was recorded of a microcrystalline mixture of at least three different jack bean proteins (urease, concanavalin A, concanavalin B). A one megapixel Adaptive Gain Integrating Pixel Detector (AGIPD) was used which has not only a high frame rate but also a large dynamic range. This dataset is publicly available through the Coherent X-ray Imaging Data Bank (CXIDB) as a resource for algorithm development and for data analysis training for prospective XFEL users.

## Abstract (summary)

**Table:** 

Design Type(s)	protocol testing objective
Measurement Type(s)	protein structure data
Technology Type(s)	x ray crystallography
Factor Type(s)	
Sample Characteristic(s)	Canavalia

Machine-accessible metadata file describing the reported data (ISA-Tab format)

## Background & Summary

The high peak brightness and femtosecond duration of X-ray pulses provided by X-ray free-electron lasers (XFELs) enable acquisition of essentially radiation damage free diffraction data^1^. This allows structure determination of highly damage-prone systems such as metalloproteins^2,3^. Moreover, XFELs afford data collection of small and/or weakly diffracting particles including microcrystals kept at room temperature. Diffraction at room temperature is of particular interest since it opens the door for time-resolved experiments^4–6^. For all these reasons beam time at XFELs is heavily oversubscribed. Thus, XFELs providing pulses at MHz repetition rate instead of 10–120 Hz, have been awaited eagerly. The European XFEL (EuXFEL) near Hamburg is the first MHz XFEL; the facility has accepted users since September 2017.

The EuXFEL has a unique pulse structure: ultimately it will deliver up to 27,000 pulses per second, organized in 10 pulse trains per second with a 4.5 MHz repetition rate within each train^7–9^. In order to make use of as many X-ray pulses as possible, fresh sample needs to be available for each pulse and the diffraction data need to be recorded and stored. This poses great challenges for fast enough sample delivery as well as detectors. The Adaptive Gain Integrating Pixel Detector (AGIPD)^10^ was developed specifically for use at EuXFEL. In addition to a fast acquisition rate (up to 3520 images/s recorded with 10 trains/s), which is achieved through an analogue memory capable of storing 352 images, and operation at 4.5 MHz frame rate^10^, the AGIPD provides a large dynamic range. This is made possible by a dynamic gain switching amplifier in each pixel. This allows for each pixel a dynamic range of more than 10^4^ 12.4 keV photons in the lowest and single photon sensitivity in the highest gain mode.

Here we describe serial femtosecond crystallography (SFX) data collected at the Single Particles, Clusters, and Biomolecules and Serial Femtosecond Crystallography (SPB/SFX) instrument^11,12^ of the European XFEL in June 2018. The goal was to establish whether there is a detrimental influence of the previous X-ray pulse on the sample probed by the following pulse. Since we could exclude this for the current experimental conditions already during the beam time using lysozyme microcrystals as a well-established model system^13^ – which was also observed in another experiment published after our beam time^14^, we decided to also investigate a previously uncharacterized sample, a mixture of microcrystals of different jack bean proteins. The present data set contains the results of these diffraction measurements. We used this data to determine the structure of two of the proteins^13^. However, we did not perform detailed checks for damage; therefore such an analysis can still be performed. Moreover, the data allow the testing of algorithms for efficient indexing of mixtures containing crystals with different unit cells. This is important if unit cell dimensions in a sample either differ due to non-isomorphism, change due to dehydration during sample delivery or due to structural changes induced by reaction initiation in time-resolved experiments. Moreover, the data allow testing algorithms for calibration of the AGIP detector, and may be used to develop and benchmark data analysis routines for data collection at EuXFEL.

## Methods

These methods are expanded versions of descriptions in our related work^13^.

### Sample preparation and injection

Proteins were extracted from jack bean meal (from Sigma (J0125)) using acetone following published procedures^15,16^. The proteins were crystallized at 4 °C as described using a batch crystallization approach^13^. After three weeks, at least three morphologically distinct kinds of microcrystals were observed with rod-, needle- and rugby ball-like shapes. The microcrystalline slurry was filtered using a 20 µm stainless steel inline filter. For injection via a liquid microjet produced by a gas dynamic virtual nozzle (GDVN) injector^17^ using helium as the focusing gas, the sample concentration was adjusted to contain 10–15% (v/v) settled crystalline material. During injection the sample was kept at 4 °C in a rotating temperature-controlled reservoir to prevent crystal settling as described in ref.^18^. The sample flow rate was 30–40 µl min^−1^, and gas pressure 400–500 psi at the inlet of the GDVN’s gas supply line, corresponding to a flow rate of 140–250 ml min^−1^. In order to reproducibly flow enough sample fast enough to close the gap created in the jet by the previous X-ray pulse^19^ in time before the next pulse arrives, the jet speed must be measured *in situ* during data collection both on a regular basis and for each change in flow conditions (e.g., new sample, crystal concentration, change in liquid flow rate or helium pressure, new GDVN, etc.). To this end the jet was imaged using a femtosecond laser to prevent blurring of the images as described recently^19^. The fs laser pulse and the camera were triggered by the EuXFEL global trigger (10 Hz) that indicates the arrival of an X-ray pulse train, thus the images were recorded at a set delay relative to the arrival of the pulse train. This delay was set so as to image the jet shortly after the second pulse generated a visible gap in the jet, thus imaging the effect of the first two pulses on the jet. Imaging two gaps in the jet that are produced by two X-ray pulses therefore allows determining jet speed in a single image. To enable comparison of all data collected in a liquid jet, jet speed was always set to a value of 40–50 m s^−1^, typically ~45 m s^−1^, by adjusting sample flow rate and pressure of the focusing gas.

### Data collection

The experiment was performed at the SPB/SFX instrument of the EuXFEL^11,12^. The accelerator was delivering ten pulse trains per second with 60 pulses per train. The first 10 pulses of each train were used for electron orbit feedback and then being sent to the pre-undulator dump, without lasing. The remaining 50 pulses of the train (1.1. MHz intra-train repetition rate; we measured 886.15 ± 0.01 ns spacing between pulses) were used for data collection. The photon energy was tuned to a nominal value of 7.48 keV. The X-ray focus was ~15 µm, electron bunch length ~50 fs FWHM. For each individual X-ray pulse, the pulse energy was recorded by an X-ray gas monitor detector (XGMD) upstream of the experimental hutch showing that each pulse had 0.9–1.5 mJ pulse energy. With a beamline transmission of ~70%, this yields a flux of up to 5.0⋅10^9^ photons/μm^2^ per pulse (9.9⋅10^22^ photons/(μm^2^ s)) at the sample position.

### Detector calibration

Details on the detector and its general calibration procedure can be found in a separate publication^20^. This section provides a brief overview of the steps included to calibrate the detector for the described experiment. The raw data, as output by the AGIP detector, was corrected and calibrated with facility-provided automatic calibration^21–23^. The calibration constants were derived by EuXFEL and the AGIPD consortium^20^, and are applied on a per-pixel, per-memory cell and per-gain mode basis.

In a first step, the gain setting for each pixel is evaluated from the digitized analogue gain information provided by the detector. Two thresholds exist, derived from dark image data, and gain settings of high, medium and low are assigned depending on whether the pixel’s gain value is below the first, between both, or above the second threshold. Subsequently, this information alongside the memory cell index is used to correct the offset/pedestal value for each pixel with the appropriate constant. Offset constants are evaluated as the median pixel value of a set of dark images and adjusted by an additional switching offset for medium and low gain stages, which was derived from pulse capacitor and charge injection data. Pulse capacitor and charge injection data is acquired in special operation modes of the detector, which use ASIC-internal current sources for signal generation without X-rays present. The additional switching offset adjustment is necessary as offsets differ slightly, depending on whether a pixel has automatically switched gain due to integrated charge, or was forced to switch into a particular gain setting (as is the case for dark image data). Finally, a relative gain correction is performed. The relative gain constants were obtained by first determining the relative slopes of the medium and low gain stages with respect to the high gain stage, using pulse capacitor and charge injection data respectively. The relative high gain of a given pixel with respect to all pixels of the detector was determined from flat field data (Cu-fluorescence), by evaluating the positions of the first five photon peaks. The high-to-medium and high-to-low gain relative slopes are then used to scale this high gain constant, providing constants for medium and low gain. All characterizations further yield bad pixel masks, which are provided on a per-image basis alongside the calibrated data, and are already selected for the appropriate gain and memory cell.

**Fig. 1:**
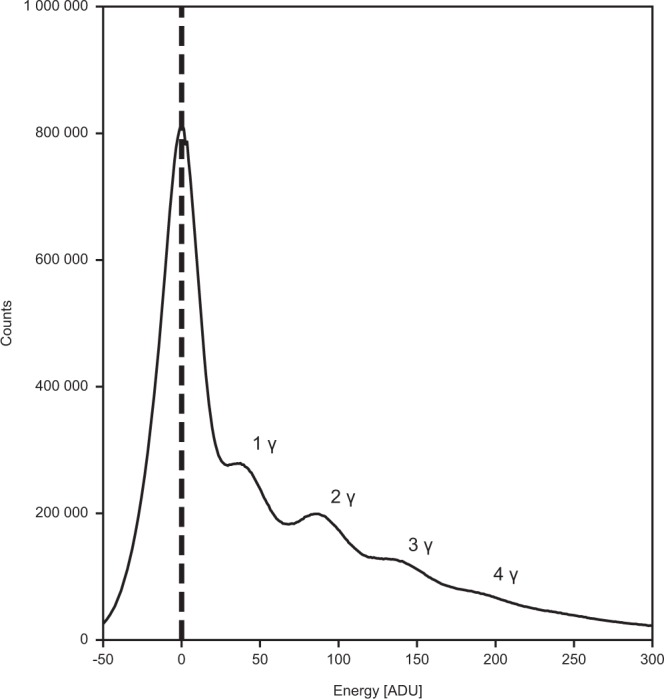
Quality of the detector calibration. A histogram of corrected data from all AGIPD detector modules and 64 X-ray pulses of data taken from run 342. The noise peak is centred at 0, indicating proper memory-cell specific offset correction. Additionally, the first 4 photon peaks can be distinguished, showing that relative gain correction was appropriate for each individual memory cell and pixel.

The quality of the detector calibration at the time of the experiment can be judged from the histogram of corrected data from all modules and 64 pulses (Fig. 1). This together with the presence of anomalous signal in the diffraction data^13^ shows that the corrections are adequate for the described experiment. They reflect the knowledge about the detector at the time of the experiment (June 2018). Since then, understanding of the detector has increased further.

### Data processing and structure solution

**Fig. 2:**
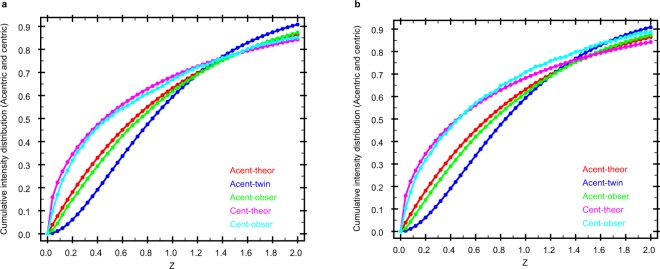
Cumulative intensity distribution of the diffraction data. The plots were calculated using TRUNCATE^38,39^ for **(a)**. Concanavalin A (PDBID: 6gw9) and **(b)**. Concanavalin B (PDBID: 6gwa).

CASS^24,25^ was used for online data analysis^23^ of the corrected detector data and offline hit identification. A hit is defined as an image where more than ten Bragg spots were identified. To this end we used the algorithm described in ref.^25^. Indexing and integration were performed with CrystFEL version 0.6.3^26^. The sample-detector distance was determined by indexing rate optimization, yielding a value of 121 mm. A nominal value of 7.47 keV was used for indexing. The position of the sample jet was continuously adjusted to maximize the hit rate. The positions and orientations of individual sensor modules of the AGIPD were refined as described^4^. Due to the large number of saturated pixels in the corrected detector images, the top and the bottom row of detector ASICs were excluded from the geometry file to prevent contamination of integrated detector signals with artefacts. In addition, three further ASICs on the right side of the detector were observed to switch off and on during data recording and thus were excluded as well (see Figure Less_panel_geom in the Auxiliary File available together with the deposited data on the Coherent X-ray Imaging Data Bank website (CXIDB)^27,28^). The concanavalin B data were subjected to AMBIGATOR to remove the indexing ambiguity^29,30^. The cumulative intensity distributions of the data agree with the theoretically expected distributions, as shown in Fig. 2. This reflects the quality of the detector calibration in general and the successful indexing of the polar space group of concanavalin B in particular.

## Data Records

In total, 1,333,750 diffraction images were collected of the microcrystalline jack bean protein mixture. The final number of indexed diffraction patterns is 76,803 for concanavalin A and 23,719 for concanavalin B, with the resolution limit of the Monte-Carlo integrated data being 2.1 Å in both cases. Data collection statistics for concanavalin A and B are listed in Table 1. We did not follow up on the urease data because of the low resolution of the data. Due to the large size of the raw data we only deposited those images identified as hits on the CXIDB^27^ website with the CXIDB ID 87^28^. During this experiment the pulse energy determined with the gas monitor detectors was not assigned to the same data location as the diffraction images (the pulse train number metadata, “trainID”, did not match). The pulse energy information per pulse was therefore not used, and was removed from the deposited data.

**Table 1:** Data collection statistics.

	Concanavalin A (6GW9)	Concanavalin B (6GWA)
Space group	*I*222	*P*6_1_
Cell dimensions		
*a*, *b*, *c* (Å)	63.9, 88.1, 90.2	82.3, 82.3, 103.4
α, β, γ (°)	90.0, 90.0, 90.0	90.0, 90.0, 120.0
Resolution (Å)	45–2.1 (2.2–2.1)^a^	42–2.2 (2.3–2.2)
*R*_split_	0.128 (0.694)	0.146 (0.560)
CC_1/2_	0.984 (0.333)	0.967 (0.232)
CC*	0.996 (0.706)	0.992 (0.614)
*I*/σ(*I*)	7.2 (2.0)	7.6 (3.2)
Completeness (%)	100.0 (100.0)	100.0 (100.0)
Multiplicity	715 (146)	723 (241)

The number of indexed crystals are 76,803 for concanavalin A and 23,719 for concanavalin B. ^a^Values in parentheses are for the highest resolution shell.

The refined structural models and integrated scaled diffraction data have been deposited in the Protein Data Bank (accession code: 6GW9 (concanavalin A)^31^ and 6GWA (concanavalin B)^32^.

$${R}_{split}=\left(1/\sqrt{2}\right)\cdot \frac{{\sum }_{hkl}\left|{I}_{hkl}^{even}\,-\,{I}_{hkl}^{odd}\right|}{\frac{1}{2}{\sum }_{hkl}\left|{I}_{hkl}^{even}+{I}_{hkl}^{odd}\right|}$$

## Technical Validation

We successfully phased the diffraction data of concanavalin A (using PDB entry 1JBC^33^ as the search model after removal of the waters and the metal ions) and concanavalin B (using 1CNV^34^ as the search model after removal of the waters) using molecular replacement with PHASER^35^. The structures were refined by iterative cycles of rebuilding in COOT^36^ and refinement using PHENIX^37^, including simulated annealing. With R_work_/R_free_ of 0.186/0.238 and 0.161/0.213 and r.m.s. deviations of 0.002 Å and 0.009 Å in bond lengths and 0.577° and 1.210° in bond angles for concanavalin A and concanavalin B, respectively, the final models have excellent geometry. Detailed refinement quality indicators can be found in ref.^13^. The overall structures are highly similar to those determined using macroscopic crystals, with core RMSDs on Cα atoms against reference structures of 0.31 Å for concanavalin A (vs. PDB entry 1JBC^33^) and 0.24 Å for concanavalin B (vs. PDB entry 1CNV^34^).

## Data Availability

Data were processed with CrystFEL 0.6.3. CrystFEL 0.6.3 is a free open source software under the GNU Public License version 3 and can be downloaded from http://www.desy.de/~twhite/crystfel/. CASS is publicly available on GitLab (https://gitlab.gwdg.de/p.lfoucar/cass). The script used to optimize detector geometry is publicly available on GitHub (https://github.com/tbarends/pygeom).

## ISA-Tab metadata

Download metadata file

## References

[1] Chapman HN, Caleman C, Timneanu N (2014). Diffraction before destruction. Phil. Trans. R. Soc. B.

[2] Hirata K (2014). Determination of damage-free crystal structure of an X-ray-sensitive protein using an XFEL. Nat. Methods.

[3] Suga M (2015). Native structure of photosystem II at 1.95 Å resolution viewed by femtosecond X-ray pulses. Nature.

[4] Barends TRM (2015). Direct observation of ultrafast collective motions in CO myoglobin upon ligand dissociation. Science.

[5] Pande K (2016). Femtosecond structural dynamics drives the trans/cis isomerization in photoactive yellow protein. Science.

[6] Stagno JR (2017). Structures of riboswitch RNA reaction states by mix-and-inject XFEL serial crystallography. Nature.

[7] Altarelli M (2011). The European X-ray free-electron laser facility in Hamburg. Nucl. Instrum. Meth. Phys. Res. B.

[8] Altarelli, M. The European X-ray Free-Electron Laser: toward an ultra-bright, high repetition-rate x-ray source. *High Power Laser Sci. Eng*. **3**, 10.1017/hpl.2015.17 (2015).

[9] Tschentscher T (2017). Photon Beam Transport and Scientific Instruments at the European XFEL. Appl. Sci..

[10] Henrich B (2011). The adaptive gain integrating pixel detector AGIPD: a detector for the European XFEL. Nucl. Instrum. Meth. Phys. Res. A.

[11] Mancuso, A. P. *Conceptual Design Report: Scientific Instrument Single Particles, Clusters, and Biomolecules (SPB*). Report No. XFEL.EU TR-2011-007, 10.3204/XFEL.EU/TR-2011-007 (2011).

[12] Mancuso, A. P., Aquila, A., Borchers, G., Giewekemeyer, K. & Reimers, N. *Technical design report: Scientific Instrument Single Particles, Clusters, and Biomolecules (SPB)*. Report No. XFEL.EU TR-2013-004, 10.3204/XFEL.EU/TR-2013-004 (2013).

[13] Grünbein ML (2018). Megahertz data collection from protein microcrystals at an X-ray free-electron laser. Nat. Commun..

[14] Wiedorn MO (2018). Megahertz serial crystallography. Nat. Commun..

[15] McPherson A, Geller J, Rich A (1974). Crystallographic studies on concanavalin B. Biochem. Biophys. Res. Commun..

[16] Jabri E, Lee MH, Hausinger RP, Karplus PA (1992). Preliminary crystallographic studies of urease from jack bean and from *Klebsiella aerogenes*. J. Mol. Biol..

[17] Weierstall U, Spence JCH, Doak RB (2012). Injector for scattering measurements on fully solvated biospecies. Rev. Sci. Instrum..

[18] Lomb L (2012). An anti-settling sample delivery instrument for serial femtosecond crystallography. J. Appl. Crystallogr..

[19] Stan CA (2016). Liquid explosions induced by X-ray laser pulses. Nat. Phys..

[20] Allahgholi, A. *et al.* The Adaptive Gain Integrating Pixel Detector at the European XFEL. *J. Synchrotron Rad*. **26** (2019). 10.1107/S1600577518016077 PMC6337892 30655470

[21] Kuster M (2014). Detectors and calibration concept for the European XFEL. Synchrotron Radiat. News.

[22] European XFEL Detector Group. *European XFEL Offline Calibration Documentation*, https://in.xfel.eu/readthedocs/docs/european-xfel-offline-calibration/en/latest/ (2018)

[23] Fangohr, H. *et al.**Data analysis support in KARABO at European XFEL*. 10.18429/JACoW-ICALEPCS2017-TUCPA01 (2018)

[24] Foucar L (2012). CASS-CFEL-ASG software suite. Comput. Phys. Commun..

[25] Foucar L (2016). CFEL–ASG Software Suite (CASS): usage for free-electron laser experiments with biological focus. J. Appl. Cryst..

[26] White TA (2012). CrystFEL: a software suite for snapshot serial crystallography. J. Appl. Crystallogr..

[27] Maia FRNC (2012). The Coherent X-ray Imaging Data Bank. Nat. Methods.

[28] Gorel, A., Foucar, L., Hilpert, M. & Roome, C.M. *Coherent X-ray Imaging Data Bank* 10.11577/1472096 (2018).

[29] White TA (2016). Recent developments in CrystFEL. J. Appl. Crystallogr..

[30] Brehm W, Diederichs K (2014). Breaking the indexing ambiguity in serial crystallography. Acta Crystallogr. D.

[31] Concanavalin A structure determined with data from the EuXFEL, the first MHz free electron laser. *Worldwide Protein Data Bank* http://identifiers.org/pdb:6GW9 (2018)

[32] Concanavalin B structure determined with data from the EuXFEL, the first MHz free electron laser. *Worldwide Protein Data Bank* http://identifiers.org/pdb:6GWA (2018).

[33] Parkin S, Rupp B, Hope H (1996). Atomic Resolution Structure of Concanavalin A at 120 K. Acta Crystallogr. D.

[34] Hennig M, Jansonius JN, Terwisscha van Scheltinga AC, Dijkstra BW, Schlesier B (1995). Crystal Structure of Concanavalin B at 1.65 Å Resolution. An “Inactivated” Chitinase from Seeds of *Canavalia ensiformis*. J. Mol. Biol..

[35] McCoy AJ (2007). Phaser crystallographic software. J. Appl. Crystallogr..

[36] Emsley P, Cowtan K (2004). *Coot*: model-building tools for molecular graphics. Acta Crystallogr. D.

[37] Adams PD (2010). PHENIX: a comprehensive Python-based system for macromolecular structure solution. Acta Crystallogr. D.

[38] French S, Wilson K (1978). On the treatment of negative intensity observations. Acta Cryst. A.

[39] Winn MD (2011). Overview of the *CCP4* suite and current developments. Acta Crystallogr. D.

